# Advances of HDAC inhibitors in tumor therapy: potential applications through immune modulation

**DOI:** 10.3389/fonc.2025.1576781

**Published:** 2025-06-27

**Authors:** Jiaqi Tian, Miaomiao Han, Fuyang Song, Yun Liu, Yuhou Shen, Jiateng Zhong

**Affiliations:** ^1^ Department of Pathology, School of Basic Medical Sciences, Xinxiang Medical University, Xinxiang, China; ^2^ Xinxiang Engineering Technology Research Center of Digestive Tumor Molecular Diagnosis, the First Affiliated Hospital of Xinxiang Medical University, Xinxiang, China; ^3^ Department of Abdominal Surgical Oncology Ward 2, Xinxiang Central Hospital, Xinxiang, China; ^4^ Henan Province Engineering Technology Research Center of Tumor Diagnostic Biomarkers and RNA Interference Drugs, The Third Affiliated Hospital of Xinxiang Medical University, Xinxiang, China

**Keywords:** HDACi, tumor therapy, immunotherapy, tumor microenvironment, combination therapy

## Abstract

Histone deacetylase inhibitors (HDAC inhibitors, HDACi) have garnered considerable attention due to their potential in treating various types of malignant tumors. Histone deacetylases (HDACs) not only influence chromatin structure and gene transcription by regulating histone acetylation status but also acetylate various non-histone proteins. They are widely involved in several key biological processes, such as cell cycle regulation, apoptosis induction, and immune responses. HDACi exert their effects by inhibiting HDAC activity; however, these effects are highly concentration-dependent and non-selective. HDACi inevitably disrupt both gene expression and signaling networks, leading to multi-target, non-specific biological effects. This article focuses on the immunomodulatory mechanisms of HDACi, including their role in remodeling the tumor extracellular matrix and their impact on various immune cell populations. The synergistic potential of combining HDACi with other therapeutic approaches is also discussed. This review examines the application of HDACi across different tumor types, highlighting preclinical and clinical evidence that demonstrates the multifunctionality and efficacy of HDACi. By leveraging their unique mechanism of action, HDACi opens new avenues for enhancing antitumor immunity and achieving durable therapeutic responses. Future research and clinical trials will play a crucial role in optimizing the use of HDACi, elucidating resistance mechanisms, and identifying the most effective combinations to maximize patient benefit.

## 1 Introduction

**Figure 1:**
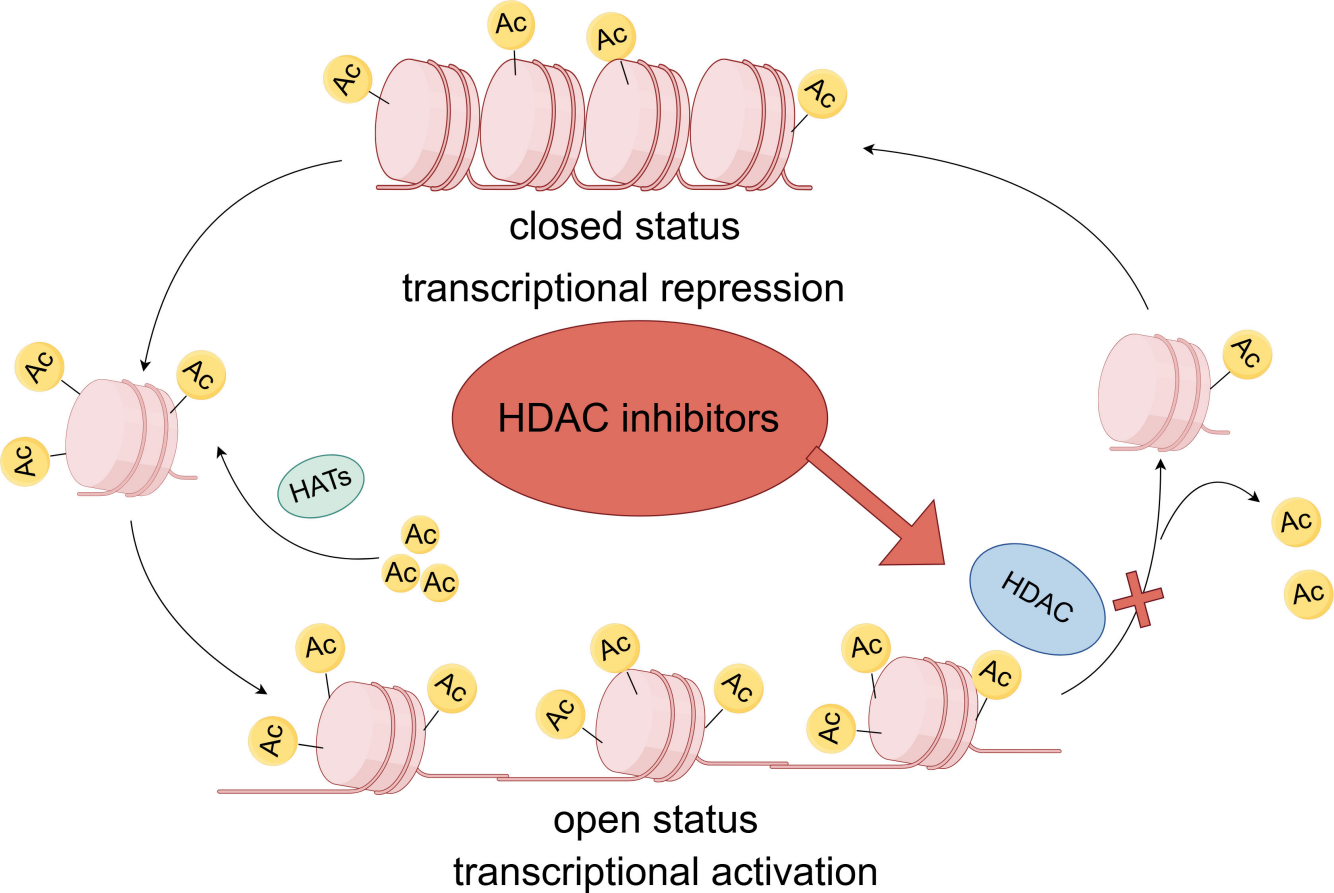
Dynamic regulatory processes of HDAC and HDACi. Chromatin consists of an association of DNA and proteins assembled in the nucleosome, the functional unit of genetic material. Nucleosomes are octamers composed of individual histone proteins. DNA is negatively charged, and histones are positively charged; therefore, the two are attracted to each other. Histone acetyltransferases(HATs) add an acetyl group to histone lysine residues. The positively charged acetyl group can neutralize the charge interaction between DNA and histones, thereby loosening the chromatin structure and facilitating transcription. In contrast, histone deacetylase removes the acetyl group from histones, reducing the degree of histone acetylation and inhibiting transcription. Inhibitors of histone deacetylase block the action of histone deacetylase, thereby increasing the degree of histone acetylation and loosening the chromatin structure, which promotes transcription.

HDACs are a crucial class of enzymes that can be categorized into four distinct classes according to homology to yeast proteins. Class I includes HDAC1, 2, 3, and 8, which are usually localized in the nucleus, have deacetylase activity, and contain zinc-dependent active sites (1, 2). Class II is further divided into class IIa (HDAC4, 5, 7 and 9) and class IIb (HDAC6 and 10), which have tissue-specific expression and can shuttle between the nucleus and the cytoplasm, and class III sirtuins (SIRT1-7) are not dependent on zinc but on NAD^+^ as a cofactor (3, 4). The only Class IV HDAC is HDAC11, which exhibits characteristics similar to those of Class I and Class II HDACs (**Table 1**) (5–7). HDACs play a central regulatory role in cellular processes by dynamically modulating the acetylation of both histones and non-histones. In the nucleus, HDACs remove acetyl groups from histones, which in turn alter chromatin structure and regulate crucial processes like gene expression, cell cycle progression, and cellular differentiation (8, 9). In the cytoplasm, HDACs also target various non-histone substrates, including transcription factors, DNA repair proteins, cytoskeletal proteins, and molecular chaperones. They regulate the function, stability, and subcellular localization of these proteins through deacetylation modifications (10–12). HDACs catalyze histone deacetylation, which increases the positive charge on histones and enhances electrostatic interactions with negatively charged DNA molecules. This epigenetic modification can cause chromatin to compact, creating a transcriptionally repressive chromatin environment (**Figure 1**) (13–15). In certain genomic regions, epigenetic reprogramming can work synergistically with regulatory elements like DNA methylation or transcription factor complexes, ultimately resulting in the transcriptional silencing of functional genes (16, 17). The range of target genes silenced by this mechanism is broad, including, but not limited to, tumor suppressor genes and pro-apoptotic genes (18, 19).

**Table 1:** Classification of HDACs based on homology to human yeast homologs and generalization of their cofactor, size, and cellular localization.

HDAC	Member	Cofactor	Size(aa)	Cellular localization
Class I	HDAC1	Zinc dependent	482	Nucleus
	HDAC2		488	Nucleus
	HDAC3		428	Nucleus
	HDAC8		377	Nucleus/Cytoplasm
Class IIa	HDAC4		1084	Nucleus/Cytoplasm
	HDAC5		1122	Nucleus/Cytoplasm
	HDAC7		952	Nucleus/Cytoplasm/Mitochondria
	HDAC9		1011	Nucleus/Cytoplasm
Class IIb	HDAC6		1215	Cytoplasm
	HDAC10		669	Cytoplasm
Class IV	HDAC11		347	Nucleus/Cytoplasm
Class III	Sirt1	NAD^+^ dependent	747	Nucleus/Cytoplasm
	Sirt2		389	Cytoplasm
	Sirt3		399	Mitochondria
	Sirt4		314	Mitochondria
	Sirt5		310	Mitochondria
	Sirt6		355	Nucleus
	Sirt7		400	Nucleus

HDAC inhibitors are a class of drugs that inhibit the activity of histone deacetylases, thereby regulating gene expression and affecting processes such as the cell cycle, cell differentiation, and apoptosis. HDAC inhibitors can be classified based on their synthetic or natural composition, subclass specificity, and chemical structure type. In general, they are divided into two categories: pan-inhibitors of HDACs and HDAC-specific inhibitors. HDAC inhibitors are classified into four main subgroups based on their chemical composition: hydroxamic acids, benzamides, cyclic tetrapeptides, and short-chain fatty acids (20). Several HDAC inhibitors, including Vorinostat, Romidepsin, Panobinostat, and Belinostat, have been approved by the U.S. Food and Drug Administration. In addition, China and Japan have approved Tucidinostat, a novel subtype-selective HDAC inhibitor that targets class I HDACs (HDAC1, HDAC2, and HDAC3) as well as class IIb HDAC10 (**Table 2**) (26). HDAC inhibitors are considered novel epigenetic drugs whose therapeutic potential has been extensively tested in various disease models. For example, Vorinostat has shown efficacy in the treatment of cutaneous T-cell lymphoma, particularly by inhibiting the activity of class I and class II HDACs. Romidepsin is capable of disrupting the G1 and G2 phases of the cell cycle and inducing apoptosis (29). Panobinostat acts as a general inhibitor of tumor cell growth by affecting misfolded protein aggregation through the inhibition of HDAC6 and the upregulation of p21 (30). Belinostat, a hydroxy acid pan-HDAC, for the treatment of multiple cancers (31). Tucidinostat acts as a selective HDAC inhibitor and affects tumor cell growth and death through the inhibition of specific HDAC subtypes (32). These drugs have shown encouraging results in the treatment of multiple myeloma, Hodgkin lymphoma, non-Hodgkin lymphoma, breast cancer, lung cancer, and many other cancers.

**Table 2:** Introduction to the mechanism of action, trial phase, and FDA approval of HDACi.

Name of drug	Mechanism of action	FDA approved	Phase of trial	Reference
Vorinostat (SAHA)	Regulation of the cell cycle	Yes	I, II	(21)
Entinostat	Altered gene expression	No	I, II, III	(22)
Romidepsin	Induction of cell cycle arrest	Yes	I, II, III	(23)
Belinostat	Preventing acetyl removal	Yes	I, II, IV	(24)
Panobinostat	Blocking the cell cycle	Yes	I, II, III	(25)
Tucidinostat (Chidamide)	Increased acetylation	No	I, I, III	(26)
Scriptaid	Inhibition of cell proliferation	No	I, II	(27)
Trametinib	Increased acetylation	Yes	I, I, III	(28)

## 2 Mechanism of action of HDACi in immunotherapy

**Figure 2:**
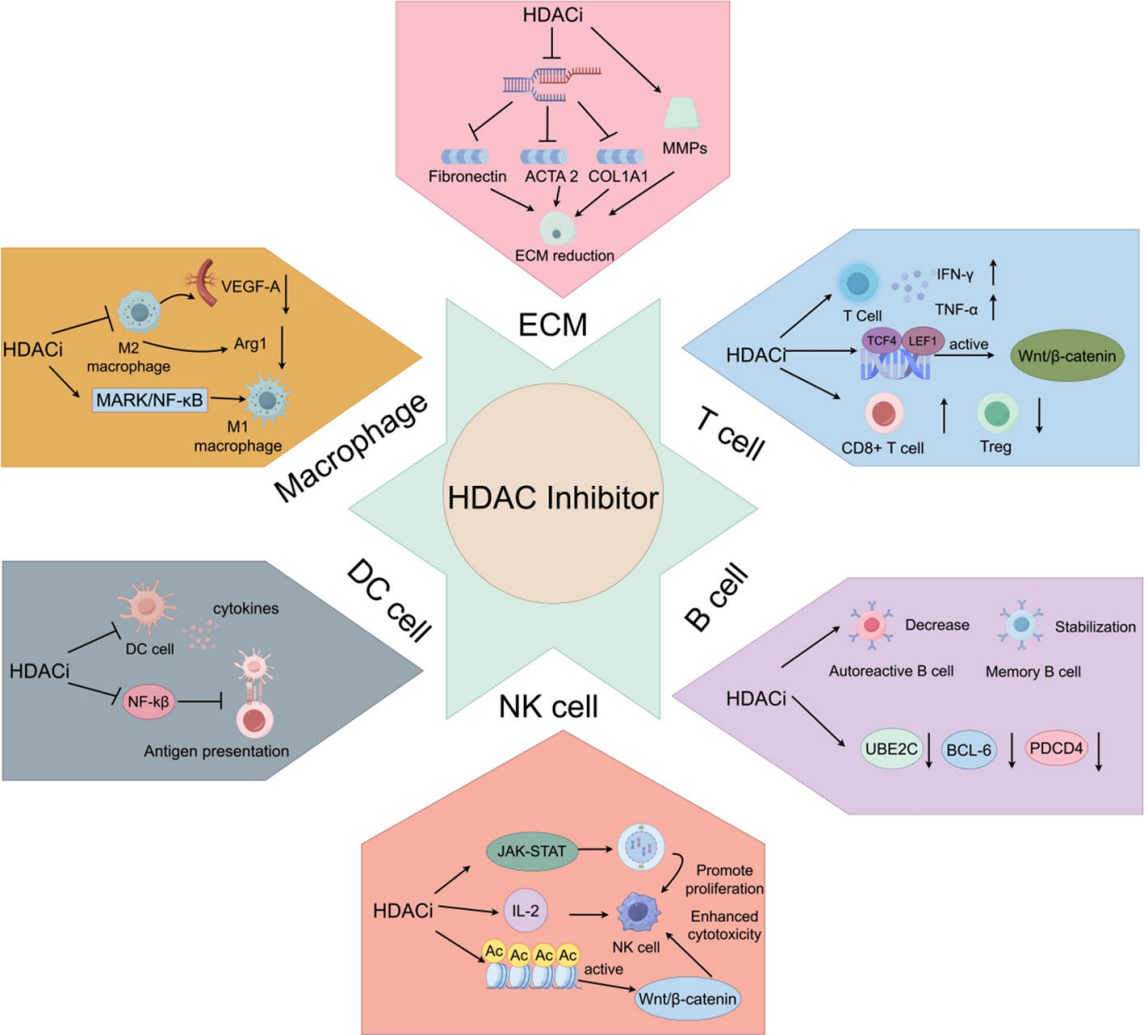
Mechanism of action of HDACi on immune cells. ECM: HDACi promotes ECM reduction. Macrophage: HDACi promotes M1 macrophage polarization and inhibits M2 macrophage polarization. T cell: HDACi activates the Wnt/β-catenin signaling pathway, promotes cytokine release, and reduces the number of Tregs. B cell: HDACi promotes the reduction of autoreactive B cells, maintains the number of memory B cells, and promotes apoptosis of malignant B cells in B-cell lymphoma. NK cell, HDACi enhances cytotoxicity and promotes cell proliferation. DC, HDACi inhibits the release of cytokines by dendritic cells and suppresses their antigen-presenting capacity by downregulating the NF-κB signaling pathway.

HDAC inhibitors exert complex and multifaceted immunomodulatory effects in a concentration-dependent manner by targeting key immune components in the tumor microenvironment (TME), including the extracellular matrix (ECM), macrophages, T cells, B cells, natural killer cells, and dendritic cells (**Figure 2**). These effects help reshape the tumor microenvironment (TME) and influence anti-tumor immune responses, offering new strategies for cancer immunotherapy (**Table 3**).

**Table 3:** Summary of the effects of HDACi on tumor stromal cells and various immune cells.

Cell Type	Representative HDACi	Administration Route and Concentration	Effect	References
Tumor stromal cells	Panobinostat, VPA	*In vitro*: 85 nM (Pano), 1.5 mM (VPA)	improves TME	(33)
	Scriptaid	*In vitro*: 10 μM	Delays tumor growth	(34)
Macrophages	SAB	*In vitro*: 20 μM	Blocks M2 polarization	(35)
	TMP195	*In vitro*: ≥40 μM; *In vivo*: 50 mg/kg	Promotes M1 polarization	(36)
T cells	MS-275	*In vivo*: 100 μg/mouse/day	Reverses T-cell exhaustion	(37)
	Romidepsin, Vorinostat	*In vitro*: 10 nM (Romi), 2 μM (Vori)	Enhances CD8^+^ T-cell cytotoxic function	(38)
	STR-V-53	*In vivo*: Combined with the PD-1 inhibitor	Downregulates Tregs proportion	(39)
B cells	Panobinostat	*In vivo*: 10→5 mg/kg/day; *In vitro*: 0.55–5 nM	Reduces autoantibodies	(40)
	Vorinostat, MS-275	*In vitro*: 2 μM (Vori), 0.2 μM (MS-275)	Suppresses humoral immunity	(41, 42)
NK cells	Panobinostat	*In vitro*: 5–20 nM	Promotes proliferation and cytotoxic activity	(43, 44)
	Entinostat	*In vitro*: 0.5 μM	Enhances IFN-γ secretion and cytotoxicity	(45)
Dendritic cells	Panobinostat	*In vitro*: 2.5–20 nM	Inhibits DC maturation and pro-inflammatory function	(46)
	Panobinostat	*In vivo*: 25 mg/kg	Activates IFN-I secretion	(47)
	TSA	*In vitro*: 20 nM	Impairs T-cell activation capacity	(48)

### 2.1 Remodeling of tumor extracellular matrix by HDACi

The extracellular matrix (ECM) is a key component of TME. HDACi has been reported to alter the structure of TEM and reduce the invasiveness of tumor cells and cancer-associated fibroblasts (CAFs) by inhibiting the expression of fibronectin (FN) and collagen. According to Martina Korfei et al. (33), treatments with Panobinostat (85 nM) and Valproic acid (VPA, a class I HDACi, 1.5 mM) decreased the mRNA expression of genes related to fibronectin, ACTA2 (actin), and COL1A1 (collagen). CAFs support tumor progression and invasion by secreting an abundant extracellular matrix that protects tumor cells from immune checkpoints or kinase inhibitors. According to Dae Joong Kim et al. (34), Scriptaid (a selective inhibitor of HDACs 1/3/8, 10 μM) inhibits ECM secretion, reduces cell contraction and rigidity, and impairs collective cell invasion in CAF and tumor cell spheroid co-cultures. Scriptaid reduces CAF abundance and delays tumor growth *in vivo*. In addition, HDACi promotes the degradation of ECM by regulating the activity of matrix metalloproteinases (MMPs), thereby attenuating the interaction between tumor cells and the stroma and further inhibiting tumor metastasis (49). These effects not only help inhibit tumor growth but also provide new strategies for immunotherapy of tumors. However, clinical trials remain indispensable for translating this strategy into clinical applications.

### 2.2 Effect of HDACi on macrophages

The role of HDACi in regulating the tumor microenvironment is receiving increasing attention, particularly its impact on tumor-associated macrophages. Macrophages can be classified into M1-type (classically activated macrophages) and M2-type(alternatively activated macrophages). IFN-γ can differentiate macrophages into pro-inflammatory M1-type macrophages (50). Unlike IFN-γ, IL-4 can convert macrophages into M2-type macrophages, which are associated with anti-inflammatory functions (51). M2-type macrophages exert synergistic effects in reducing inflammatory responses, promoting tissue repair, enhancing immune suppression, and supporting tumor growth by secreting anti-inflammatory cytokines (IL-10 and TGF-β) and highly expressing arginase-1 (Arg1) (52). Trametinib (HDACi) inhibits M2-type polarization of macrophages, leading to a reduction in the expression of pro-angiogenic growth factors VEGF-A and Arg1 in these cells (53). SAB (20 μM, HDAC10 inhibitor) significantly inhibits the gene expression of M2 macrophage polarization markers (Arg1, Fizz1, Ym1) in both *in vitro* models (35). Yicheng Han et al. (36) found that TMP195 (HDACi) can reprogram macrophages into an M1 phenotype at specific concentrations (50 mg/kg *in vivo* and ≥40 μM *in vitro*), thereby enhancing the antitumor immune response. In addition, TMP195 continuously activates MAPK and NF-κB signaling pathway phosphorylation, suggesting that it promotes M1 polarization through the synergistic action of epigenetic modification and inflammatory signaling pathways (54). In summary, HDAC inhibitors exert immune regulatory effects by modulating the polarization balance of macrophages.

### 2.3 Regulation of T cell function by HDACi

HDAC inhibitors play an important role in regulating T cell function. Exhausted T cells exhibit high expression of inhibitory receptors, such as PD-1 and TIM-3, and a loss of effector function (55, 56). To overcome this bottleneck, Andrew Nguyen’s team (37) conducted a study using Class I HDAC inhibitor MS-275 (100 μg/mouse/day) and found that MS-275 downregulated the expression of the exhaustion marker PD-1, thereby relieving its functional inhibition, while upregulating TIM-3. However, its expression exhibited dynamic decoupling from exhaustion characteristics. In contrast, terminal effector differentiation markers (KLRG1 and granzyme B) were significantly enriched. The viral activator Tax plays a crucial role in HTLV-1 reactivation and the initiation of new infections. Annika P. Schnell et al. (57) demonstrated that Panobinostat (100 nM) and Romidepsin (10 nM) significantly increased Tax transcription levels in CD4^+^ Jurkat T cells. Mohammed L. Ibrahim et al. (38) conducted a study *in vitro* with HDAC inhibitors at specific concentrations (10 nM Romidepsin or 2 μM Vorinostat). They observed a dose-dependent increase in TNF-α and IFN-γ secretion. Real-time cytotoxicity analysis confirmed that HDACi significantly enhances the cytotoxic activity of CD8^+^ T cells against tumor target cells. HDACi can also activate the Wnt/β-catenin signaling pathway by upregulating the expression of specific transcription factors, such as TCF4 and LEF1, thereby enhancing the antitumor activity of T cells (58). Preclinical studies have demonstrated that the novel hepatocellular carcinoma-selective HDAC inhibitor STR-V-53 not only inhibits HDAC activity and upregulates PD-L1 expression in tumor cells but also significantly enhances CD8^+^ T cell infiltration. Notably, the combination of STR-V-53 with a PD-1 inhibitor synergistically reduces the proportion of regulatory T cells (Tregs) (39). This contrasts with the mechanism of HDACi (SAHA) found in previous studies. SAHA can induce the generation of Tregs in the thymus of mice, promote the conversion of peripheral T cells to Tregs (59). We speculate that this apparent contradiction may stem from differences in the subtype selectivity and effective concentrations of HDAC inhibitors. This suggests a significant association between HDAC subtype-specific inhibition and immune regulatory effects.

### 2.4 Regulation of B cell function by HDACi

The primary function of B cells is to produce antibodies and mediate humoral immune responses. They can also present soluble antigens and produce cytokines, participating in immune regulation (60). When administered via intraperitoneal injection to lupus-prone mice, the HDAC inhibitor Panobinostat significantly reduced the number of autoreactive plasma cells and serum antinuclear antibody levels. Importantly, this treatment did not affect the number of CD4^+^/CD8^+^ T cells or spleen weight. Further *in vitro* experiments confirmed that Panobinostat, within the low nanomolar concentration range (0.55–5.00 nM), selectively inhibited B cell proliferation and differentiation. However, it did not impair the activity of memory B cells (40). Depletion of certain key immune cell types during the early inflammatory phase has been shown to prevent sodium sulfate-induced colitis (DSS colitis). Studies have found that the HDAC6 inhibitor BML-281 does not affect the infiltration of neutrophils, macrophages, or T lymphocytes in DSS-treated mice. Still, it does inhibit the infiltration of CD191^+^ B lymphocytes into the inflamed lamina propria (61). B-cell antibody class-switch recombination (CSR) and somatic hypermutation (SHM) depend on the functional activity of activation-induced cytidine deaminase (AID) and uracil-DNA glycosylase (UNG2) (62). Studies have shown that HDACi affects CSR and SHM by reducing UNG2 expression levels. Following HDACi treatment, the expression levels of the cell cycle-related protein UBE2C and the anti-apoptotic protein BCL6 significantly decreased, while the expression level of the pro-apoptotic protein PDCD4 significantly increased. HDACi directly induces apoptosis of malignant B cells in B-cell lymphoma by increasing pro-apoptotic proteins (41). René Winkler et al. (42) found that HDACi (0.2 μM MS-275, 1.5 mM Valproic acid) increased H3/H4 acetylation levels by inhibiting histone deacetylase activity, interfering with chromatin openness and DNA repair complex recruitment, thereby significantly inhibiting B cell CSR and reducing the expression of IgG subclasses (IgG1, IgG2a) and IgA. HDAC inhibitors regulate B cell function through epigenetic reprogramming, exhibiting dual antitumor effects. Further exploration is needed to optimize dosage and precisely regulate combination therapy strategies.

### 2.5 Role of HDACi in enhancing natural killer cell activity

Natural killer (NK) cells are crucial immune effector cells that can limit the expansion and spread of cancer cells. Studies have shown that HDACi can enhance the killing ability of NK cells against tumor cells by promoting their proliferation and activation. Low concentrations (5 nM) of HDACi Dactinostat and Panobinostat can significantly enhance the proliferation of primary natural killer cells (pNK cells) induced by IL-2 through the JAK-STAT signaling pathway (43). Panobinostat (10–20 nM) activates the Wnt/β-catenin signaling pathway by increasing the level of histone H3K27 acetylation in the β-catenin promoter region, thereby significantly upregulating the expression of NKG2D ligands MICA and MICB on the surface of soft tissue sarcoma cells, and subsequently enhancing the killing efficiency of NK92 cells and pNK cells against tumor cells (44). The treatment with 0.5 μM Entinostat for 24 hours significantly upregulates the expression of NKG2D, simultaneously inducing increased chromatin accessibility to activate IFIT1 gene transcription and its downstream STING-STAT4-IRF1 signaling pathway. Functional experiments confirmed that Entinostat pre-treatment of NK cells enhances their cytotoxic activity against tumors and IFN-γ secretion capacity (45). However, Lucas E. Rossi et al.’s findings contrast sharply with the previous view. They studied peripheral blood NK cells from healthy donors and NK cells from the spleens of C57BL/6 mice. Their results showed that HDAC inhibitors (0.1–1 μM Trichostatin A (TSA); 2–10 mM Valproic acid) significantly inhibited IL-12/IL-15/IL-18-induced IFN-γ secretion and the cytotoxic activity of NK cells (63). This contradictory effect may be related to the dose gradient, treatment duration, and cell type specificity of HDACi, suggesting that the administration regimen of HDACi needs to be precisely optimized in clinical applications to achieve positive regulation of NK cell function.

### 2.6 Effect of HDACi on dendritic cells

Dendritic cells (DCs) are key antigen-presenting cells in the immune system, playing a critical role in antitumor immunity (64). It was assessed whether HDACi could modulate cytokine production by DCs. During the maturation process induced by lipopolysaccharide (LPS) or poly (I: C) (polyinosinic-polycytidylic acid), DCs were treated with or without Panobinostat at concentrations of 2.5, 10, and 20 nM for 24 hours. Compared to the control, Panobinostat treatment significantly inhibited the production of a range of cytokines by DCs, including IL-6, IL-10, IL-12, IL-23, and TNF-α (46). The HDAC inhibitor Panobinostat (25 mg/kg) activates IFN-I secretion by increasing H3K27 acetylation of type I interferon genes in pDCs (plasma cell-like dendritic cells) (47). HDACi was shown to inhibit the maturation process of dendritic cells, thereby affecting their ability to present antigens. Trichostatin A (HDACi, 20 nM) was found to inhibit the maturation of dendritic cells by down-regulating the NF-κB signaling pathway, thereby decreasing their ability to stimulate T cells (48). Additionally, HDACi may also influence the role of DCs in the tumor microenvironment by altering their cellular phenotype and secretory profile. This inhibitory effect may lead to increased immunosuppression in the tumor microenvironment, which in turn affects the effectiveness of the overall immune response (65, 66). Based on the above, we find that the effect of HDACi on DCs appears to be more harmful than beneficial.

## 3 Joint application of HDACi

HDAC inhibitors significantly enhance the antitumor efficacy of immune checkpoint inhibitors, CAR-T therapy, and chemotherapy through epigenetic remodeling. Specifically, they synergistically activate antitumor immune responses, enhance the long-term functionality of CAR-T cells, promote chemotherapy drug sensitivity, and mitigate treatment toxicity (**Table 4**). Future research should focus on exploring subtype-specific mechanisms, clinical translation pathways, and combination strategies for expanding their application in solid tumors.

**Table 4:** Summary of concentrations/doses and mechanisms for HDACi combination therapy.

Combination Therapy	Concentration/Dose	Mechanism of Action	References
SAHA + B7x neutralizing antibody	SAHA: 40 mg/kg; B7x antibody: 200 μg/mouse	Increased infiltration of CD8^+^/CD4^+^ T cells in the tumor microenvironment	(67)
BEBT-908 + Anti-PD-1 antibody	BEBT-908: 25–100 mg/kg; Anti-PD-1 antibody: 200 μg/dose	Enhanced antitumor activity	(68)
LMK235 (HDAC5 inhibitor) + Anti-PD-1 antibody	LMK235: dose not specified; Anti-PD-1 antibody: 200 μg/dose	Increased CD8^+^ T cell infiltration, decreased Tregs proportion	(69)
M344/Chidamide + CAR-T	M344: 200 nM; Chidamide: 1 μM (*in vitro*), 10 mg/kg (*in vivo*)	Activation of Wnt/β-catenin pathway	(70)
VPA + CAR-T	VPA: 0.5–1 mM (*in vitro*), 100 mg/kg (*in vivo*)	Enhanced CAR-T cytotoxicity	(71)
Valproic acid (VPA) + Cisplatin	VPA: 4 mM (*in vitro*)	Induction of cell cycle arrest and apoptosis	(72)
Vorinostat/Valproic acid + Idarubicin	Vorinostat: 0.075–1 μM; Valproic acid: 0.25–3 mM	Activation of the DNA damage response pathway	(73)

### 3.1 HDACi and immune checkpoints

Immune checkpoints are a group of key factors expressed on immune cells that regulate the level of immune activation. A key function of immune checkpoint molecules is similar to a car’s “braking system.” They can quickly intervene when the immune system is activated, acting as a timely brake. This helps ensure that immune system activation remains within moderate limits, thereby preventing excessive activation (74). Tumor cells can evade immune system surveillance by exploiting immune checkpoints, thereby allowing them to survive and proliferate. Research indicates that HDACi can enhance the efficacy of immune checkpoint inhibitors by modulating the tumor microenvironment and amplifying antitumor immune responses (75, 76). B7x is an immune checkpoint modulator. The combination therapy of SAHA (40 mg/kg, intraperitoneal injection every 3 days) and B7x neutralizing antibody (200 μg/mouse, intraperitoneal injection every 2 days) significantly delayed tumor growth, with a tumor inhibition rate of 80.6%, and significantly increased the infiltration of CD8^+^ and CD4^+^ T cells in the tumor microenvironment (67). The large amount of butyrate produced by Clostridium difficile nucleic acid in tumors inhibits HDAC3/8 in CD8^+^ T cells, inducing acetylation and expression of H3K27 in the Tbx21 promoter, thereby inhibiting PD-1, alleviating CD8^+^ T cell exhaustion, and promoting effector function (77). Tumor growth experiments conducted in syngeneic mice inoculated with MC38 cells using PD-1 immune checkpoint inhibitors demonstrated that while BEBT-908 (a dual HDAC/PI3K inhibitor) alone could delay the growth of MC38 tumors, its efficacy was significantly enhanced when combined with anti-PD-1 antibodies, with some host mice surviving for extended periods. In fact, after combination therapy, 5 out of 8 mice achieved a durable cure (68). Researchers utilized the KPC genetically engineered mouse PDAC model (a pancreatic cancer model expressing mutant KRAS and p53 under the Pdx1-Cre drive) to investigate the combined therapy of HDAC5 inhibitor LMK235 (administered intraperitoneally) and anti-PD-1 antibody (200 μg per dose, administered intraperitoneally). The results showed that inhibiting HDAC5 significantly increased CD8^+^ T cell infiltration in the tumor microenvironment, reduced the proportion of Tregs, and exhibited synergistic effects with anti-PD-1 antibodies, jointly inhibiting tumor growth (69). Combination therapy with HDACi and immune checkpoint inhibitors has shown promise in multiple clinical trials, particularly in patients with refractory tumors, where the combination therapy regimen can significantly improve patient survival rates and disease control rates (78–80).

### 3.2 Combination of HDACi and CAR-T cell therapy

In recent years, HDAC inhibitors have emerged as a promising adjuvant therapy with the potential to enhance CAR-T cell function (81). *In vitro* experiments showed that CAR-T cells pretreated with HDAC inhibitors (200 nM M344 or 1 μM Chidamide) exhibited reduced HDAC1 expression and significantly increased histone H3K27 acetylation. In *in vivo* experiments, using a leukemia mouse model, it was confirmed that HDACi pretreatment or oral administration of Citarabine (10 mg/kg) after infusion significantly reduced tumor burden and prolonged survival. The experiment also demonstrated through multi-omics analysis that HDACi activates the classical Wnt/β-catenin signaling pathway, jointly driving CAR-T cell differentiation toward a central memory phenotype and inhibiting the expression of exhaustion-related receptors (70). Valproic acid enhances CAR-T cell recognition of tumor targets and cytotoxic effects by upregulating the expression of NKG2D ligands (ULBP1-3) on tumor cell surfaces and activating the NKG2D-NKG2DL immune recognition axis (71). Additionally, HDACi can promote the secretion of cytokines such as IL-2 and IFN-γ, thereby enhancing T cell proliferation and effector functions (82, 83). However, these findings are based on preclinical experiments, and further clinical trials are necessary for future applications.

### 3.3 HDACi and chemotherapy

The application of HDACi in chemotherapy is gaining increasing attention, particularly due to their potential to enhance antitumor activity. HDACi can improve the efficacy of chemotherapeutic agents by altering the epigenetic state of tumor cells, thereby promoting cell cycle arrest and inducing apoptosis (84). For example, following Valproic acid treatment, the levels of p16, p21, and p27 were observed to increase by twofold, indicating that tumor suppressor-mediated cell cycle arrest and the observed cell death effects are involved. The Valproic acid pretreatment regimen opens chromatin, increases the expression of tumor suppressor genes, and enhances the binding of cisplatin to chromatin, ultimately leading to increased cell death (72). In gastric cancer cells, pretreatment with HDACi significantly increases the amount of DNA-binding drugs, promotes histone acetylation, and leads to cell cycle arrest and cell death (20). Studies have shown that the anthracycline drug idarubicin, when used in combination with histone deacetylase inhibitors (such as Vorinostat 0.075–1 μM or Valproic acid 0.25–3 mM), significantly enhances synergistic antitumor activity against MOLT4 and HL60 leukemia cell lines and primary acute leukemia patient cells. The mechanism of action involves the simultaneous activation of histone H3/H4 acetylation modifications and the γH2AX-mediated DNA damage response pathway, and this synergistic effect is not affected by the timing of administration (73). However, further *in vivo* experiments and toxicity assessments of normal hematopoietic stem cells are needed to determine their clinical translational value. Although chemotherapy is an important means of treating cancer, it is often accompanied by severe side effects. Research has shown that HDACi can alleviate patients suffering by improving gastrointestinal toxicity caused by chemotherapy (85). In addition, the study also found that HDACi, such as Curcumin, can alleviate chemotherapy-induced nephrotoxicity and cardiotoxicity, which may be related to their antioxidant properties (86). By combining HDACi with chemotherapy drugs, it may be possible to significantly improve patients’ quality of life and reduce the overall toxicity burden of chemotherapy without compromising antitumor efficacy (87). Therefore, the use of HDACi helps enhance the safety of chemotherapy, making it an important adjuvant drug in cancer treatment.

## 4 HDAC inhibitors in oncology

**Figure 3:**
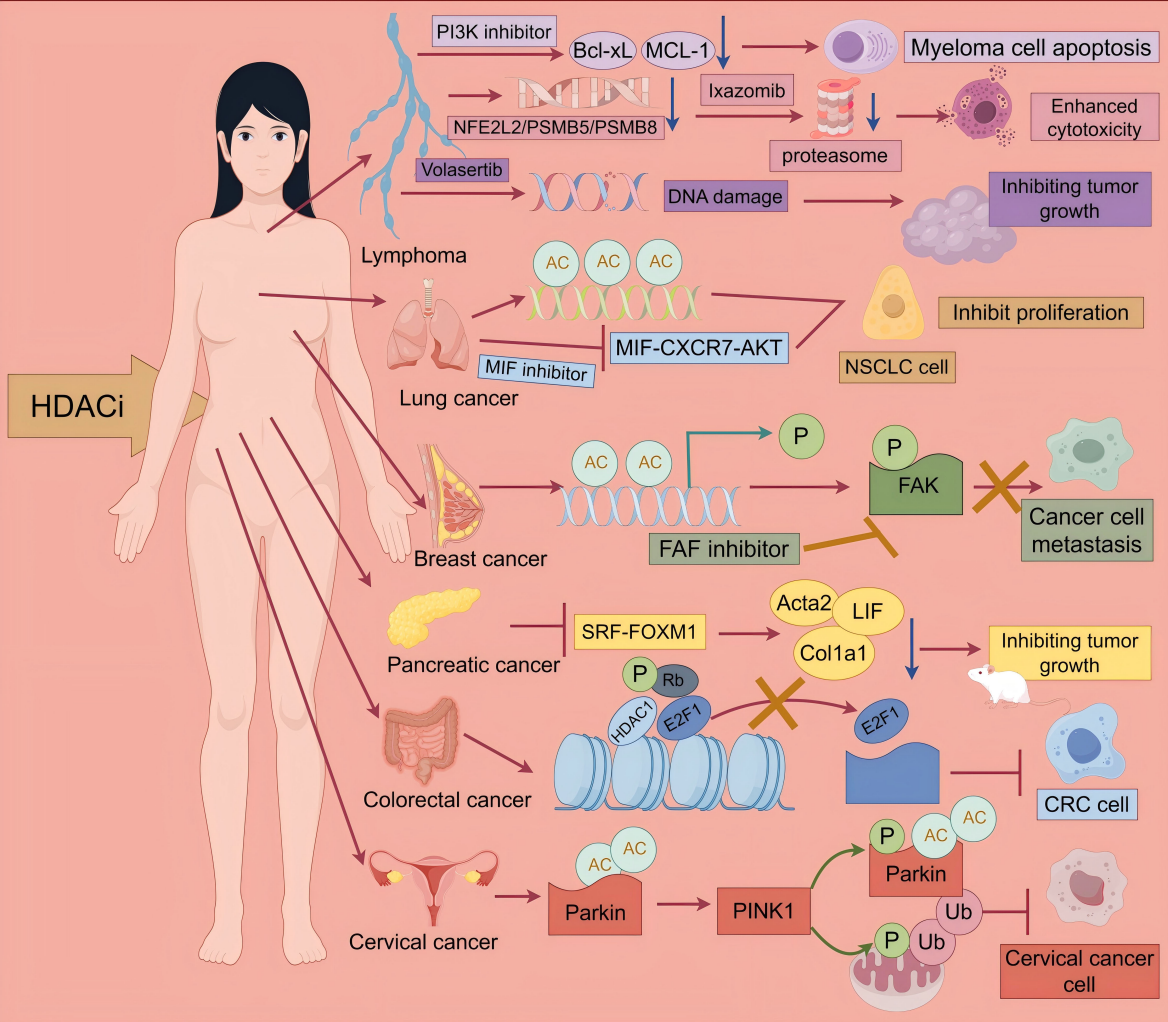
Antitumor effects of HDACi. multiple myeloma: Multiple myeloma: CUDC-907 simultaneously targets HDAC and PI3K, reducing the expression of Bcl-xL and MCL-1, thereby inhibiting myeloma cell growth. Hodgkin lymphoma: Ixazomib combined with Belinostat can synergistically inhibit the NFE2L2 pathway, suppress proteasome activity, and thereby enhance cytotoxicity. Non-Hodgkin lymphoma: Volasertib combined with HDACi induces DNA damage, thereby inhibiting tumor growth. Lung cancer: 6a simultaneously targets HDAC and MIF, inducing histone acetylation and blocking the MIF-CXCR7-AKT pathway, thereby inhibiting the proliferation of NSCLC cells. Breast cancer: HDACi can promote metastasis by enhancing H3K9 acetylation of the NEDD9 gene promoter, upregulating NEDD9 expression, and activating FAK phosphorylation. FAK inhibitors can reverse this process. Pancreatic cancer: HDACi inhibits the SRF-FOXM1 transcriptional axis, downregulating the pro-fibrotic genes Acta2 and Col1a1 and the pro-inflammatory factor LIF, thereby inhibiting tumor growth. Colorectal cancer: HDACi reduces the phosphorylation level of Rb, preventing E2F1 from being released from the E2F1/Rb/HDAC1 complex. The retention of E2F1 prevents it from activating downstream target genes that drive the cell cycle process, ultimately inhibiting the growth of CRC cells. Cervical cancer, HDACi activates PINK1/Parkin-mediated mitochondrial autophagy, thereby inhibiting the growth of cervical cancer cells.

Histone deacetylase inhibitors demonstrate significant potential in the treatment of various hematologic malignancies and solid tumors. They exert their antitumor effects through multiple mechanisms, including epigenetic modification, inhibition of key signaling pathways, and modulation of the tumor microenvironment (**Figure 3**). Clinical studies across different phases have provided preliminary evidence supporting their clinical therapeutic value (**Table 5**). Future research efforts should focus on optimizing therapeutic strategies, exploring combination regimens, and developing precision biomarkers aimed at enhancing efficacy and reducing toxicity.

**Table 5:** Summary of the efficacy of HDACi in clinical trials for various tumors. PO indicates oral administration, IV indicates intravenous injection.

Cancer Type	Treatment Regimen and Dosage	Clinical Stage	Clinical Efficacy	Reference
Multiple Myeloma	Carfilzomib (36 mg/m² IV, twice weekly) + Panobinostat (20 mg PO, three times weekly; 3 weeks on/1 week off)	Phase I	ORR 63%, median OS 23 months; thrombocytopenia as primary toxicity	(88)
	Bisthianostat monotherapy (100→200→400 mg PO, twice weekly)	Phase Ia	Disease stabilisation in 50% of patients	(89)
Hodgkin Lymphoma	Vorinostat (300 mg/d PO) + Sirolimus (4 mg/d PO)	Phase I	ORR 55% (CR 27%), median PFS 5.8 months	(90)
Non-Hodgkin Lymphoma	Tucidinostat (20 mg PO, Days 1/4/8/11 of 21-day cycle) + RCHOP	Phase II	Complete response rate 86% in treatment-naive high-risk DLBCL	(91)
	Panobinostat (15–20 mg PO, three times weekly) + Everolimus (7.5–10 mg/d PO)	Phase II	ORR 25%, but 100% of patients experienced Grade 3/4 thrombocytopenia	(92)
	Abexinostat monotherapy (45 mg/m² PO, twice daily ×7 days intermittent dosing)	Phase I/II	Relapsed Follicular Lymphoma: ORR 64.3%, median PFS 20.5 months	(93)
Breast Cancer	Vorinostat (300 mg/d PO) + Ixabepilone (Regimen B: 16 mg/m² IV)	Phase IB	Regimen B: ORR 30%, CBR 35%, median PFS 3.7 months, median OS 17.1 months; ≥Grade 3 neurotoxicity 21%	(94)
Lung Cancer	Vorinostat (400 mg/d PO) + Pembrolizumab (200 mg IV every 3 weeks)	Phase I/IB	Disease control rate 67%; significant clinical benefit in patients with high CD8^+^ T-cell density	(95)
	Entinostat (10 mg PO, once weekly in two doses) + Erlotinib (150 mg/d PO)	Phase II	Significantly prolonged median OS in the E-cadherin high expression subgroup	(96)
Pancreatic Cancer	Entinostat (5 mg/week PO) + Nivolumab (240 mg IV every 2 weeks)	Phase II	Median PFS 1.89 months; ≥Grade 3 toxicity rate 63%	(97)
Colorectal Cancer	Chidamide (30 mg PO, twice weekly) + Sintilimab (200 mg IV every 3 weeks) + Bevacizumab (7.5 mg/kg IV every 3 weeks)	Phase II	ORR 44.0%; median PFS 7.3 months; ORR 50% in liver metastasis subgroup	(98)

### 4.1 Multiple myeloma

In recent years, HDACi has shown significant potential in the treatment of multiple myeloma (MM) through epigenetic regulation, induction of apoptosis, and inhibition of key pathways (PI3K/Akt/mTOR and NF-κB). Studies have shown that the dual inhibitor CUDC-907 (10 nM) effectively inhibits myeloma cell growth and downregulates anti-apoptotic proteins (Bcl-xL, MCL-1) at lower concentrations by simultaneously targeting HDAC and PI3K. Its efficacy is significantly superior to that of Vorinostat (HDAC inhibitor) or Pictilisib (PI3K inhibitor) at 1 μM (99, 100). In a clinical study, a phase I trial of carfilzomib (intravenous infusion, dose range 27–45 mg/m²) in combination with palbociclib (oral, dose range 15–20 mg) confirmed the feasibility of the non-glucocorticoid combination regimen. This dose group achieved an objective response rate (ORR) of 63% and a median overall survival (OS) of 23 months; however, hematological toxicities, such as thrombocytopenia, were observed (88). The Phase I trial of the novel HDAC inhibitor Bisthianostat (BIS, oral tablets, starting dose of 100 mg, escalated to 200/400 mg, administered twice weekly) demonstrated good safety as a monotherapy, with no Grade 3/4 non-hematologic toxicities. Fifty percent of patients achieved stable disease (SD), including one case in the 200 mg group that maintained SD for nearly seven months, providing a solid foundation for future therapies (89).

### 4.2 Hodgkin lymphoma

Hodgkin lymphoma (HL) is a specific type of lymphoma that is typically treated with chemotherapy and radiation therapy, but HL cells often develop resistance to traditional treatments. Frank C. Passero Jr. et al. (101) found that in Hodgkin lymphoma cell lines, the proteasome inhibitor Ixazomib (25–75 nM) transiently inhibits proteasome activity, but subsequent functional recovery occurs due to NFE2L2 (NRF2)-dependent upregulation of proteasome gene expression. In contrast, the HDAC inhibitor Belinostat (250 nmol/l) blocked this adaptive response by downregulating NFE2L2 and proteasome genes (PSMB5, PSMB8). A phase I trial involving 40 patients with relapsed/refractory HL that the combination of Vorinostat (300 mg/day orally) with sirolimus (4 mg/day orally, n=22) or Everolimus (5-10 mg/day dose escalation, n=18) yielded ORR of 55% and 33%, respectively, with a median progression-free survival (PFS) of 5.8 months, suggesting synergistic efficacy of HDACi combined with mTOR inhibitors (90). Although current data support the notion that HDACi overcomes resistance through epigenetic regulation and inhibition of pro-survival pathways, the limitations of small sample sizes and non-randomized designs necessitate validation through larger-scale studies (102). Future research should explore precision combination strategies based on biomarkers, such as NFE2L2, to optimize treatment for relapsed/refractory HL (103).

### 4.3 Non-Hodgkin lymphoma

Multiple studies have demonstrated that the efficacy and toxicity of HDACi in treating non-Hodgkin lymphoma (NHL) are highly dependent on the administration strategy and disease subtype. In preclinical studies, the combination of PLK1 inhibitor Volasertib (intravenous injection, 30 mg/kg) and HDACi Belinostat (intraperitoneal injection, 50 mg/kg) significantly inhibited tumor growth in a DLBCL/MCL mouse model by synergistically inducing DNA damage and downregulating c-Myc (104). Clinical translational research shows that in a Phase II clinical trial of newly diagnosed high-risk diffuse large B-cell lymphoma (DLBCL) (n=100), oral HDAC inhibitor Tucidinostat (20 mg per dose) in combination with the R-CHOP regimen achieved an 86% complete response rate (91). A phase II clinical trial in relapsed/refractory DLBCL evaluating the combination of the oral HDAC inhibitor palbociclib (40 mg three times weekly) with the oral mTOR inhibitor Everolimus showed an objective response rate of 25%. However, the regimen demonstrated limited clinical utility due to universal grade 3/4 hematologic toxicity, with thrombocytopenia occurring in 83% of patients (92). A multicenter phase I/II trial showed significant efficacy of oral pan-HDAC inhibitor Abexinostat (45 mg/m² twice daily, 7 days on/intermittent dosing) in relapsed follicular lymphoma, with a 64.3% ORR and 20.5-month median PFS. However, the response rate is limited in mantle cell lymphoma (ORR 27.3%) (93). The above results highlight the need for a comprehensive evaluation of the clinical value of HDACi in combination with specific disease subtypes and precise dosing regimens (route, dose, and cycle).

### 4.4 Breast cancer

In breast cancer, combination therapy strategies demonstrate considerable potential for overcoming the limitations associated with single-agent therapy. HDAC inhibitors can enhance H3K9 acetylation at the NEDD9 gene promoter, upregulate NEDD9 expression, and activate FAK phosphorylation, thereby promoting metastasis. Notably, preclinical evidence suggests that FAK inhibitors can reverse HDAC inhibitor-induced NEDD9-dependent metastasis, implying the feasibility of a targeted combination strategy (105). The HDAC inhibitor (SAHA) suppresses the proliferation, invasion, and migration of breast cancer cells by upregulating miR-200c to inhibit CRKL protein expression (106, 107). A randomized Phase IB clinical trial evaluated the feasibility of combining the HDAC inhibitor Vorinostat with the microtubule stabilizer ixabepilone for the treatment of previously treated metastatic breast cancer (94). Comparing two combination regimens (A and B), Regimen B, which employed a 28-day cycle with split-dose oral Vorinostat (days 1–7 and 15–21) and dose-dense, lower-dose ixabepilone (days 2, 9, and 16), demonstrated superior clinical efficacy compared to Regimen A (21-day cycle). Regimen B showed advantages in ORR, clinical benefit rate (CBR), and OS over Regimen A. While PFS was similar between the two regimens, Regimen B significantly improved patient survival. Regarding safety, the incidence of ≥ grade 3 peripheral sensory neuropathy for both regimens was significantly lower than historically reported rates for ixabepilone monotherapy or its combination with capecitabine. In summary, HDAC inhibitors demonstrate multiple mechanisms of action in breast cancer treatment. Future studies should focus on optimizing their use in combination therapy to enhance clinical efficacy (108).

### 4.5 Lung cancer

In the treatment of lung cancer, HDAC inhibitors have also shown promising application prospects. A novel HDAC/MIF dual-target inhibitor (6a) at a concentration of 12.5 μM promotes apoptosis by inhibiting HDAC activity and inducing histone acetylation (H3K27ac), while simultaneously blocking the MIF-CXCR7-AKT signaling pathway, thereby synergistically inhibiting the survival and proliferation of NSCLC cells (especially EGFR-mutant/TKI-resistant strains). Combination with TKI drugs further enhances efficacy, offering a potential dual-targeted therapeutic strategy to overcome NSCLC resistance (109). In addition, when used in combination with other chemotherapy drugs, Chidamide can effectively inhibit the proliferation of lung cancer cells and induce their apoptosis (110). Although HDACi face challenges in clinical application, such as poor water solubility, rapid clearance, and high systemic toxicity, their effectiveness in inhibiting tumor growth and metastasis has been supported by numerous studies (111). In a Phase 1/1b trial, oral HDAC inhibitor Vorinostat (400 mg/day) combined with intravenous PD-1 inhibitor pembrolizumab (200 mg every 3 weeks) was administered to 33 patients, with good tolerability and no dose-limiting toxicity. The disease control rate reached 67%, demonstrating antitumor activity in 58% of patients who had failed prior ICI therapy (95). Another Phase II randomized controlled trial evaluated the combination of oral HDAC inhibitor Entinostat (10 mg once weekly, administered in two divided doses) with Erlotinib (150 mg daily) in 132 patients with advanced NSCLC who had failed chemotherapy. Although overall efficacy did not improve, the median overall survival (OS) was significantly prolonged in the subgroup with high E-cadherin expression. The above studies screened potential beneficiary populations through biomarkers (CD8^+^ T cells or E-cadherin), providing a basis for overcoming drug resistance and developing precision treatment strategies (96).

### 4.6 Pancreatic cancer

Pancreatic cancer is one of the most aggressive solid tumors, and its treatment poses significant challenges. HDAC inhibitors have shown potential in pancreatic cancer research. Entinostat (Ent), as a Class I HDAC inhibitor, downregulates pro-fibrotic genes Acta2 and Col1a1, as well as the pro-inflammatory factor LIF, by inhibiting the SRF-FOXM1 transcriptional axis at concentrations of 5–10 μM *in vitro*, thereby blocking CAF activation and weakening the STAT3 pathway in tumor cells (112). In a mouse model, oral administration of the drug alone (5–10 mg/kg/day) reduced tumor burden by 41%. Combination therapy with gemcitabine further enhanced efficacy, with the mechanism involving increased lipogenic fibroblasts and reduced myofibroblasts to achieve “dual-chamber targeting.” Ent also attenuates the effects of TGF-β and TGF-α and antagonizes transcriptional activation (113). Another study proposed a triple epigenetic therapy combining subtoxic doses of HDAC inhibitors (Panobinostat: 7–15 nM; Vorinostat: 0.5–2 μM), a PARP inhibitor, and the demethylating agent Decitabine. The therapy overcame the traditional reliance of PARP inhibitors on BRCA gene status for inducing resistance by synergistically activating apoptotic pathways, exacerbating DNA damage responses, and dually inhibiting the DNA repair system (114). The novel HDAC inhibitor AES-135 can selectively kill pancreatic cancer cells *in vitro* without causing significant toxicity to surrounding cancer-associated fibroblasts. Additionally, AES-135 significantly prolongs survival time in mouse models, indicating its broad potential for application in the treatment of pancreatic cancer (115). In a Phase II trial of patients with metastatic pancreatic ductal adenocarcinoma (PDA) refractory to prior therapy, combination therapy with oral Entinostat (5 mg weekly) and intravenous Nivolumab (240 mg every 2 weeks) yielded partial responses in 11.1% (3/27) of patients, with response durations reaching 10.2 months. However, the regimen demonstrated a median PFS of only 1.89 months, indicating rapid disease progression in most patients. Treatment-related adverse events of grade ≥3 occurred in 63% of participants, predominantly lymphocytopenia and anemia (97). HDAC inhibitors demonstrate multi-dimensional antitumor mechanisms in pancreatic cancer, but clinical efficacy is limited by tumor heterogeneity and treatment toxicity.

### 4.7 Cervical cancer

Cervical cancer, one of the most common malignant tumors among women worldwide, has limited efficacy with current treatment strategies, necessitating the development of novel therapies. Research revealed that SAHA (Vorinostat; 1.0, 2.5, 5.0 μM) activates PINK1/Parkin-mediated mitophagy. It upregulates full-length PINK1 protein expression and enhances Parkin Ser65 phosphorylation in a time-dependent manner in Parkin-expressing HeLa cells. This indicates that SAHA promotes ubiquitination and clearance of damaged mitochondria via the PINK1-Parkin axis, thereby suppressing tumor growth (116). SAHA directly forms hydrogen bonds with the ubiquitin-conjugating enzyme UBE2C, enabling it to target and regulate the ubiquitination pathway. More importantly, in a cervical cancer mouse model, administration of SAHA significantly inhibited tumor growth without causing significant toxicity. This study reveals that by targeting the UBE2C-ubiquitination axis, SAHA coordinates the regulation of protein degradation and the epithelial-mesenchymal transition (EMT) process (117). Class I histone deacetylase inhibitor 4SC-202 exerts its anti-cervical cancer effects by targeting the prolactin receptor (PRLR) signaling pathway, thereby suppressing cancer cell proliferation and promoting cancer cell apoptosis. Importantly, *in vivo* studies confirm that oral administration of 4SC-202 significantly inhibits tumor growth and reduces PRLR pathway activity within the tumor tissue, with no observed significant hepatotoxicity or organ pathological damage, highlighting its excellent *in vivo* safety profile (118). Although research demonstrates that HDAC inhibitors effectively suppress the development and progression of cervical cancer, relevant clinical studies are still lacking. Therefore, future efforts must prioritize advancing clinical translation studies to validate their therapeutic value and optimize treatment strategies.

### 4.8 Colorectal cancer

Colorectal cancer (CRC) is one of the most common malignant tumors worldwide, with its incidence and mortality rates gradually increasing in recent years. According to statistics, in many countries, the incidence of colorectal cancer ranks second only to breast cancer and lung cancer (119). *In vitro* studies of colorectal cancer have demonstrated that HDACi effectively inhibits cancer cell proliferation and induces apoptosis. For example, the novel HDACi KH16 exhibits significant cell cycle arrest and apoptotic effects in colorectal cancer cells (120). Researchers found that HR488B (HDACi) induces cell cycle G0/G1 arrest and apoptosis through mitochondrial dysfunction, reactive oxygen species (ROS) generation, and accumulation of DNA damage, thereby specifically inhibiting the growth of CRC cells. Mechanistically, HR488B significantly reduces the phosphorylation level of the retinoblastoma protein (Rb). This prevents the release of E2F1 from the E2F1/Rb/HDAC1 complex. By sequestering E2F1 within the complex, HR488B blocks its ability to activate downstream target genes that drive cell cycle progression. Ultimately, this inhibits the growth of CRC cells (121). HDAC inhibitors have a significant regulatory effect on the characteristics of cancer stem cells (CSCs) in CRC. Research indicates that tumor stem cells possess self-renewal capacity and differentiation potential, which are key factors contributing to tumor recurrence and drug resistance. HDACi inhibit CSC proliferation and self-renewal capacity by altering histone acetylation status and influencing the expression of CSC-related genes (122). A randomized Phase II clinical trial confirmed that the triple therapy combination of an anti-PD-1 antibody (Sintilimab), an HDAC inhibitor (Citarinostat), and an anti-VEGF antibody (Bevacizumab) demonstrated significant clinical efficacy in colorectal cancer. The triple therapy group showed significantly higher objective response rates (44.0% vs. 13.0%), 18-week progression-free survival rates (64.0% vs. 21.7%), and longer median progression-free survival (7.3 vs. 1.5 months) compared to the control group. Notably, this regimen represents a breakthrough in treating patients with liver metastases, achieving a 50.0% objective response rate in this subgroup (98). HDACi monotherapy and combination immunotherapy represent a breakthrough for CRC. Future efforts should focus on optimizing combination regimens, unravelling resistance mechanisms, and developing precise biomarkers to advance the clinical translation of epigenetic therapy in the CRC field.

## 5 Conclusions and outlook

Conventional HDACi is associated with drug resistance, toxicity, and limited efficacy in clinical applications, making the development of novel HDACi an urgent issue. In recent years, researchers have begun to explore new strategies such as dual-acting HDAC inhibitors, selective HDAC inhibitors, and covalent inhibitors with the aim of overcoming the limitations of existing HDACi. For example, hydroxamic acid mixtures such as Fimepinostat and Tinostamustine have shown promising antitumor effects (123). In addition, inhibitors targeting specific subtypes, such as HDAC11, are being discovered. And the selectivity of these inhibitors may lead to fewer side effects and higher therapeutic efficacy (124). The development of novel HDACi is not limited to cancer treatment, but also extends to neurodegenerative diseases, inflammatory diseases, and other fields, showing a wide range of application prospects (125). Although this review summarizes the mechanisms of action of HDAC inhibitors in antitumor immunity and their significant achievements in laboratory and early clinical studies, substantial challenges and uncertainties persist in the clinical setting. These challenges primarily include selectivity variations among different HDAC inhibitors, determination of optimal dosing regimens, and exploration of optimal combination strategies. Current research evidence indicates that distinct types of HDAC inhibitors may demonstrate differential therapeutic effects across various cancer types. Therefore, future research must urgently focus on elucidating the mechanisms of action of HDAC inhibitors and establishing their optimal clinical application strategies.
